# Barriers and Enablers Affecting Successful Implementation of the Electronic Health Service Sisom: Multicenter Study of Child Participation in Pediatric Care

**DOI:** 10.2196/14271

**Published:** 2019-11-15

**Authors:** Petra Svedberg, Susann Arvidsson, Ingrid Larsson, Ing-Marie Carlsson, Jens M Nygren

**Affiliations:** 1 Halmstad University Halmstad Sweden

**Keywords:** children, pediatrics, eHealth, health care, quality improvement, diffusion of innovation, implementation science, participatory medicine

## Abstract

**Background:**

Children’s participation in health care is one of the most important components in the management of their disease. Electronic health (eHealth) services that are adapted to the needs of children have the potential for restructuring how children and professionals work together. Therefore, a digital interactive assessment and communication tool, Sisom, was developed to give children aged between 6 and 12 years a voice in their own health care. However, the implementation of eHealth services such as Sisom in daily practice in pediatric health care is rarely investigated.

**Objective:**

The aim of this study was to explore the process of implementing Sisom for children in pediatric care in Sweden. More specifically, the study aimed to (1) evaluate whether the implementation strategy was conducted as planned, (2) understand the barriers and facilitators of the implementation strategy in pediatric care settings, (3) gain insight into how professionals work with the specific intervention, and (4) gain insight into the usefulness and effects of the intervention from the professionals’ perspectives.

**Methods:**

A process evaluation design was used to study the implementation of Sisom at 4 pediatric care centers in Sweden. An extensive amount of qualitative and quantitative data was collected before, during, and after the intervention through self-report checklists, memos, and interviews with professionals. In total, 46 children, aged between 6 and 13 years, participated. The children used Sisom on two occasions during 6 months. When they used Sisom, a printed report formed the basis for a forthcoming dialogue between professionals, children, and their parents.

**Results:**

To our knowledge, this is the first implementation study of an eHealth communication tool aimed at strengthening children’s participation in pediatric health care. Key factors for successful implementation were alignment of the solution with the values and goals of the organization, health care professionals’ beliefs in the usefulness and usability of the solution, and health care professionals’ willingness to change their professional roles guided by the solution.

**Conclusions:**

The results from the study show that it is possible to restructure health care delivery toward a child-centered approach, if there is a willingness and preparedness in the organization to implement an eHealth solution with the aim of restructuring the way of working with children’s participation.

## Introduction

According to the United Nation’s Convention of the Rights of the Child (UNCRC), all children, regardless of gender, age, background, and disability, have the right to be heard in all matters concerning them [1]. When the UNCRC will be incorporated in Swedish law starting in January 2020, this right will apply to society in general, as well as to the child’s everyday life and when they need health care [2]. Moreover, the Swedish Patient Act [3] aims to strengthen the patient’s position through participation in their own health care. In spite of a general acceptance concerning the importance of participation, challenges remain in translating such ambitions into practice, especially when it comes to children [4]. Furthermore, the development and implementation of initiatives that promote children’s participation in health care is an underdeveloped area [5,6].

### Background

Children’s participation in health care is one of the most important components in the management of their disease and can have positive effects on the communication among children, parents, and professionals and for participation in decision processes [7]. However, their ability to participate in their own care needs to be strengthened [8-13]. It is largely limited by their restricted ability to convey their needs, expectations, and values and that communication channels focus on the health care provider and the parent [14]. This can result in increased fears and anxieties, reduced self-esteem, depersonalization, and feeling of being unprepared for procedures [15]. It is therefore important to improve the involvement of children in their own health care at a level commensurate with their experience, age, and abilities. Digital communication tools that are adapted to the needs of children have the potential to restructure how children and professionals work together by facilitating children’s opportunities and capabilities to describe their experiences and preferences and supporting their role in goal formulation and decision making [13,16]. Despite the potential of such tools, research on their implementation in clinical practice has been given less attention [5,8,17].

The use of electronic health (eHealth) services for health issues in general in pediatric health care is scarce, and few eHealth services focus on enabling and supporting children and young people’s participation in pediatric health care. Most of the eHealth services that have been developed are related to children with mental illnesses [18-20], blood disorder [21], or cancer [22] and are primarily focusing on symptom assessment, medication adherence, information, training, and self-management. We have only found one review of eHealth services that was designed to support the communication between children and health professionals with the overall purpose to strengthen children’s and young people’s participation in health care [22]. Furthermore, the evidence base to guide policy and practice for eHealth services targeting children’s participation in health issues is insufficient [19-21,23-26].

Sisom is an eHealth service [27] that was developed to give children aged between 6 and 12 years *a voice* to support their involvement in their own health care [16,28,29]. Previous research conducted on Sisom has mainly focused on issues in the design and pretesting phases around the development and evaluation of content validity, usability, and concurrent validity [15,29-35]. However, the implementation of Sisom in daily practice in pediatric health care has never been investigated. To narrow the gap between research and application in clinical practice, a thorough understanding of the implementation processes of Sisom in clinical practice is needed. This project deals with the implementation of Sisom for children in pediatric health care with the overall goal of strengthening their participation in their own health care. The implementation of research results in clinical practice is, however, in general, a challenging process, [36] and the evidence base for eHealth services is modest [37]. Therefore, the results from this study will provide valuable knowledge and guidance for policy and practice for general implementation of eHealth services in pediatric health care.

### Aim

The aim of this study was to explore the implementation process of the eHealth service Sisom for children in pediatric care in Sweden. More specifically, the process evaluation attempts to (1) evaluate whether the implementation strategy was conducted as planned, (2) understand the barriers and facilitators of the implementation strategy in pediatric care settings, (3) gain insight into how professionals work with the specific intervention, and (4) gain insight into the usefulness and effects of the intervention from the professionals’ perspectives.

## Methods

### Design

**Figure 1:**
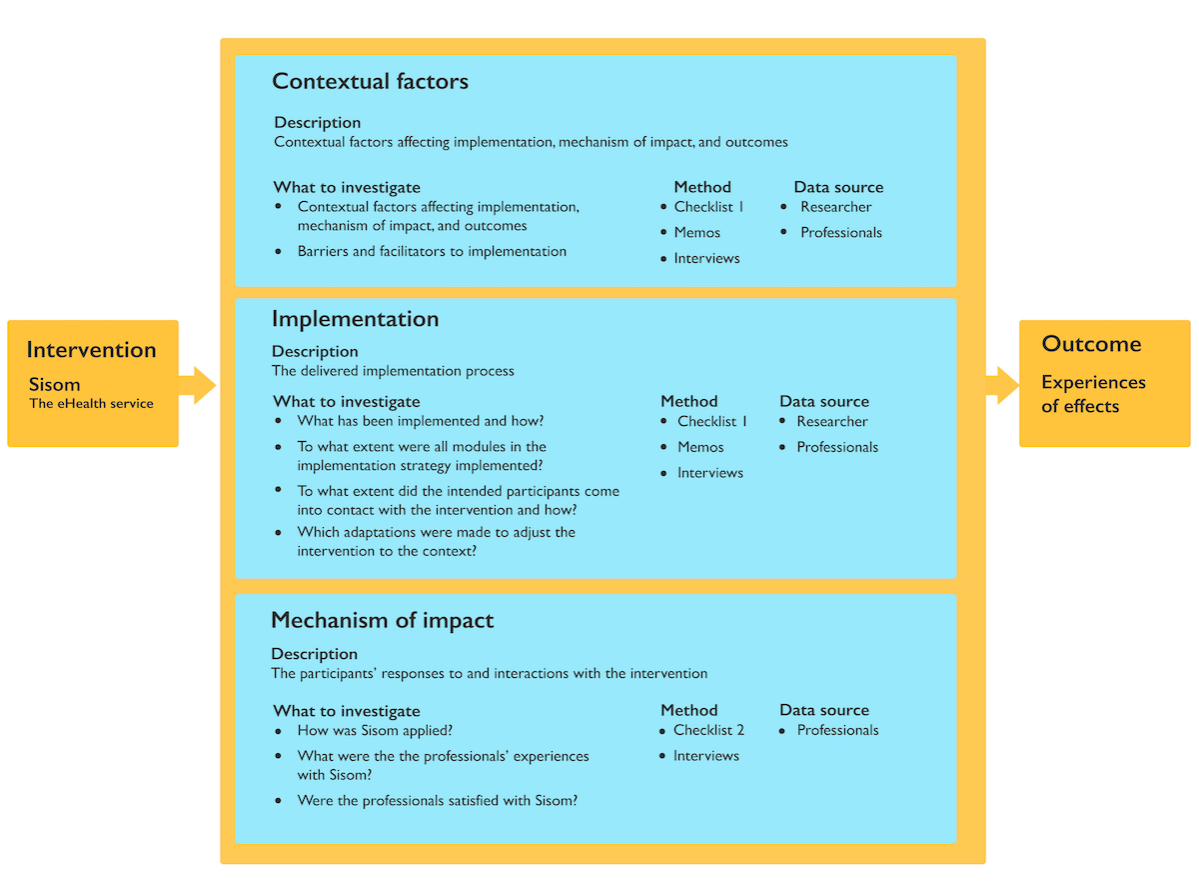
Model describing the process evaluation blueprint. eHealth: electronic health.

The implementation of an eHealth service for children in health care was studied through a process evaluation design (Figure 1) using both quantitative and qualitative methods [38]. All procedures in the study were performed with permission from the Regional Ethical Review Board in the south of Sweden (Reg. No. 2015/31).

### Study Setting and Participants

A total of 4 pediatric care centers at 3 different hospitals in Sweden were included in the study during 2016-2017. The pediatric care centers differed somewhat in terms of hospital size, care delivery processes, and the range of diagnoses treated at the center (Table 1).

**Table 1:** Study setting and participants.

Centers	Type of care	Patient group	Participants
Center A (large university hospital)	Counselor at the out- and inpatient care centers working with advice and support to facilitate everyday life for families with children with chronic illness.^a^	Children (aged 0-17 years) mostly treated for hematologic diseases and HIV infections	3 counselors
Center B (large university hospital)	Counselor at the out- and inpatient care centers working with advice and support to facilitate everyday life for families with children with chronic illness.^a^	Children (aged 0-17 years) mostly treated for various forms of cancer, diabetes, and heart diseases	10 counselors
Center C (small rural hospital)	Pediatric oncology outpatient care center with a team of pediatricians, nurses, counselors, physiotherapists, and dieticians	Children (aged 0-17 years) mostly treated for various forms of cancer	2 nurses
Center D (small rural hospital)	Pediatric neurology outpatient care center with a team of pediatricians, nurses, counselors, physiotherapists, and dieticians	Children (aged 0-17 years) treated for neurologic diseases	2 nurses

^a^At the outpatient care centers, the children usually only met the counselors, but sometimes additional professionals from the team were involved during the appointment. At the inpatient care centers, the counselors were part of a team of professionals, including pediatricians, nurses, physiotherapists, occupational therapists, psychologists, dieticians, and pedagogues.

### The Electronic Health Service, Sisom

**Figure 2:**
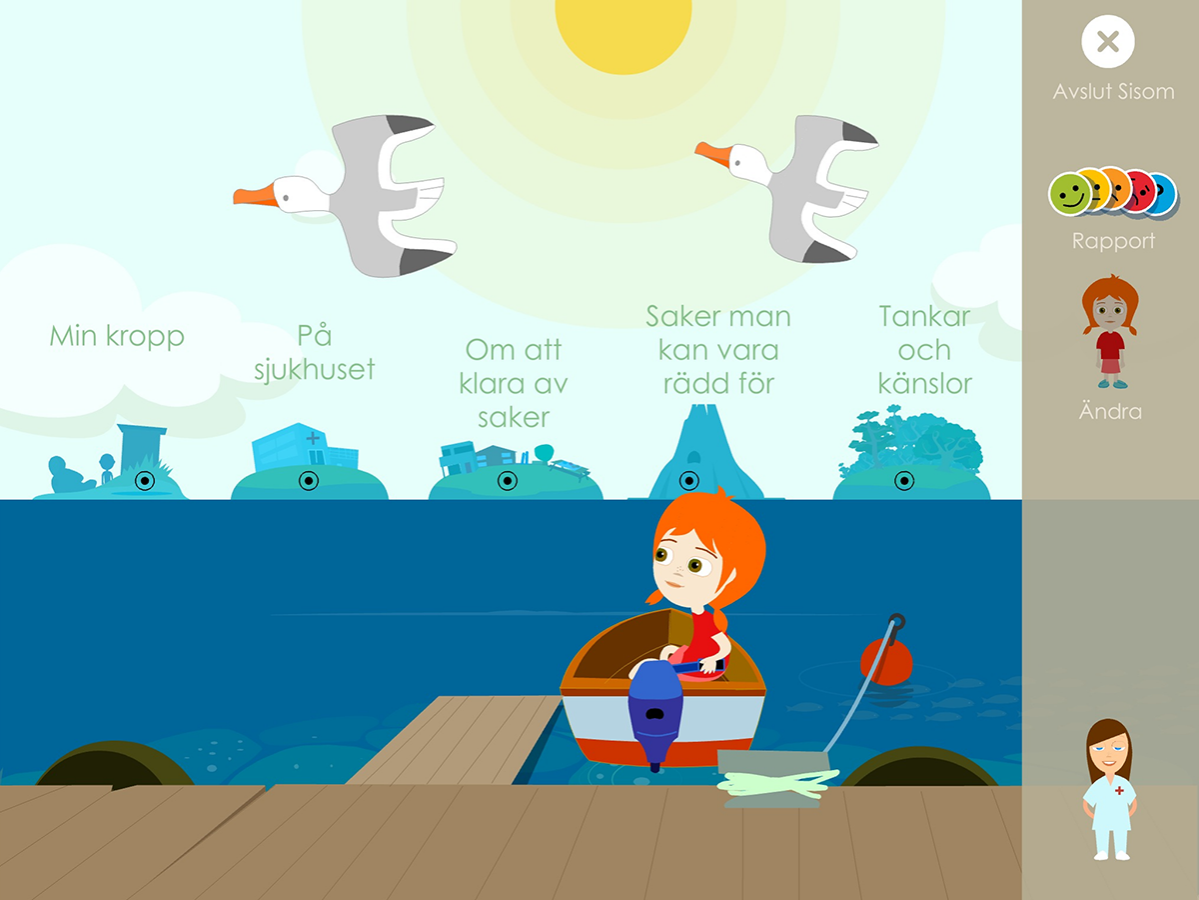
Overview of the electronic health service Sisom.

The intervention was based on an eHealth service for children in pediatric care called Sisom (Norwegian acronym for *Tell it how it is*). Sisom was developed with and for children aged between 6 and 12 years with the purpose of giving them a *voice* in their own care [16,29]. With its child-friendly interface on mobile devices, Sisom uses spoken language, text, sounds, animations, and intuitively meaningful metaphors and pictures to represent different life situations and symptoms, allowing a wide range of children to understand the meaning and communicate. In the form of a self-designed avatar, the child goes on a virtual journey traveling from island to island. Sisom currently consists of 5 islands: at the hospital, about managing things, my body, thoughts and feelings, and things one can be afraid of (Figure 2). The islands contain a total of 84 questions related to dimensions relevant to describing the children’s life situation and symptoms. The questions are presented in both text and verbally by the system. Thus, if any children had difficulties reading the text, they are guided by the audio recording of the questions and instructions. Children report their self-assessments and feelings in relation to these dimensions by selecting the level of agreement on a 5-point Likert scale with cartoon faces (with different colors and expressions). In addition, the children can specify areas of pain, bruises, and rash on a body map. When the children have gone through all the islands in Sisom, a report is printed out with the answers to the various questions. This report then forms the basis for a forthcoming dialogue between professionals and children (and sometimes parents).

### Implementation Strategy

The implementation strategy used consists of different components to support the implementation and maintenance of the eHealth service in the participating pediatric centers. The strategy was administered in the form of a 12-month program including (1) the introduction and anchoring of the project via physical meetings and email correspondence, (2) a 1-day workshop, (3) intervention period, (4) continuous facilitation, and (5) continuous support from the university, and finally (6) follow-up seminars (Table 2). In this project, the role of the local facilitators was to support and communicate with the professionals working with the intervention at each center to facilitate implementation [39].

**Table 2:** Content of the implementation strategy.

Implementation strategy components	Content	Time
Introducing and anchoring via physical meetings, email, and phone	Introducing managers and professionals to the project: discussion about how to ideally organize the project; introduction to the concept of child participation; presentation of facilitator’s role; preparation of written information to professionals and managers.	2-6 months before the workshop
Half-day or one-day workshop educating professionals and facilitators	Workshop on evidence on child participation in health care; introduction to the concept of child participation; introduction and practicing using Sisom; discussion on contextual barriers and facilitators; introducing the data collection.	Starting point
Intervention	Children and professionals use Sisom during a period of 6 months.	1-6 months
Continuous facilitation	Local facilitators support professionals in the implementation.	1-6 months
Continuous supervision	Supervisors from the university support professionals in the implementation.	1-6 months
Follow-up dialogue	Dialogue with professionals providing opportunities to share experiences of using Sisom and to discuss contextual barriers and facilitators.	1-2 months after the workshop

### Data Collection

An extensive amount of qualitative and quantitative data was collected before, during, and after the intervention through self-report checklists and interviews with professionals. The researchers also wrote memos when they were in contact with the centers and used these as data for analysis.

A total of 2 different self-report checklists were used during the project: after the education day (checklist 1) and directly after using Sisom with the child (checklist 2). The research team developed these checklists based on the components in the blueprint (Figure 1) to capture context, fidelity, dose delivered and reach, as well as professionals’ compliance with the content of the education and how they worked with the intervention.

Individual interviews or interviews in pairs were performed with 14 of the 17 professionals participating in the intervention and 1 manager at the university hospital. A total of 4 researchers conducted interviews with the participants and only with participants who they had not previously met and from centers where they had not previously participated in the implementation process. A semistructured open-ended interview guide was developed to encourage the participants to speak openly about their experiences (related to the questions in Figure 1). The interviews were audio recorded and transcribed verbatim.

### Data Analysis

Transcribed interviews, memos, and free-text responses from checklists 1 and 2 were analyzed using qualitative content analysis [40]. All transcripts were first read several times and deductively coded (by 2 of the authors) into the process evaluation components: contextual factors, implementation, mechanism of impact, and outcome [38]. An inductive coding followed, organizing the preliminary codes in potential subcategories. The analytical process was discussed continuously within the research team to increase the trustworthiness and rigor of the analysis. The data derived from checklist 2 were analyzed using descriptive statistics in SPSS 25.0 (IBM).

### Ethical Considerations

The Regional Ethical Review Board approved the study (Reg. No.: 2015/31), and the study conforms to the principles outlined in the Declaration of Helsinki [41]. All participants received written and verbal information about the study. The participants were given information about the voluntary nature of the study, confidentiality, and that they were free to withdraw their consent at any time during the process. The participants gave written consent to participate and were free to choose the time and place of the interviews.

## Results

**Figure 3:**
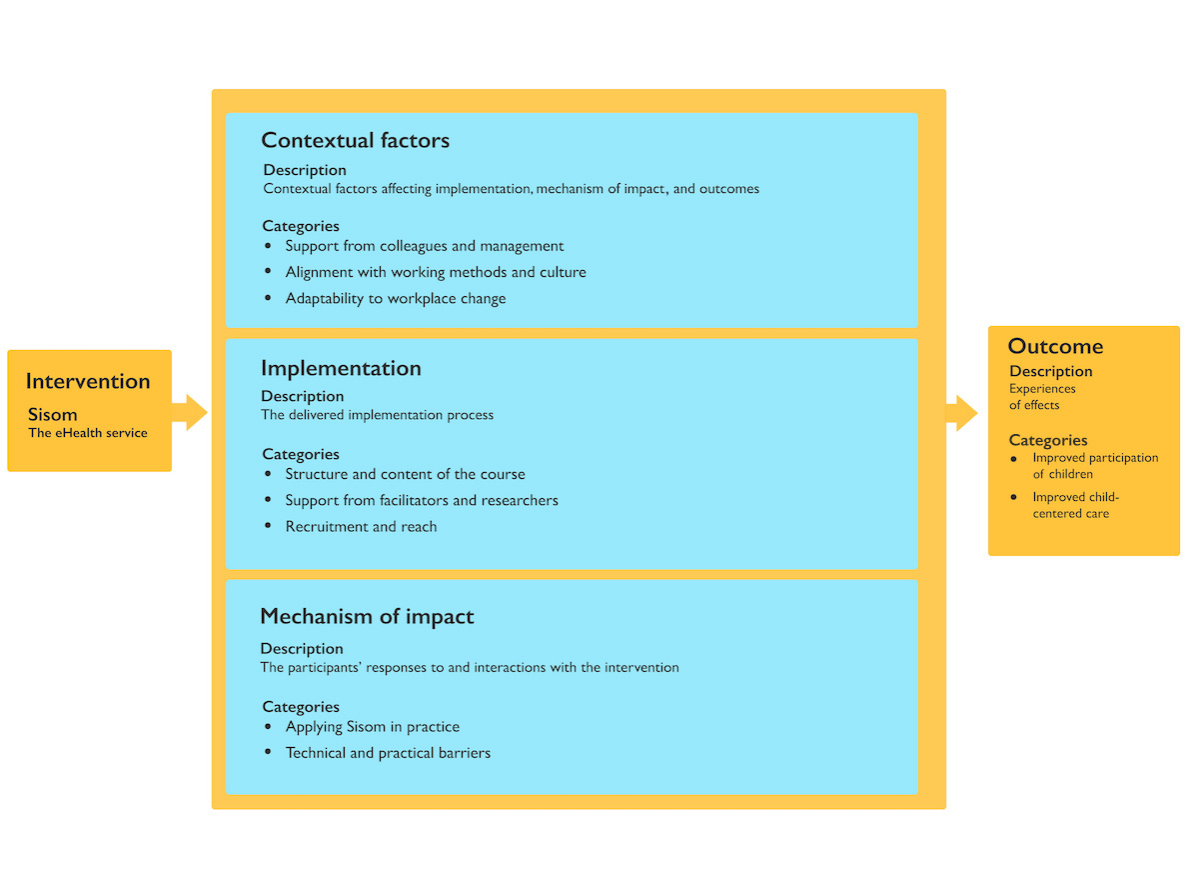
A model describing the categories and subcategories resulting from analysis of data acquired during and after implementation of Sisom according to the process evaluation blueprint. eHealth: electronic health.

The process evaluation contained 4 key components according to the evaluation blueprint: contextual factors, implementation, mechanisms of impact, and experiences of effects (Figure 3).

### Contextual Factors

A total of 3 subcategories emerged from the data: *support from colleagues and management*, *alignment with working methods and culture,* and *adaptability to* *workplace change*.

#### Support From Colleagues and Management

A decisive factor for implementing Sisom at the centers was described as the management's permission and support for carrying out the project. All participants experienced that they had support from the management to implement the project, although anchorage varied between the centers. One challenge was that hospitals often have many ongoing projects, which results in competition for resources and prioritization between projects. However, in this project, the management saw the project as part of quality development in relation to strengthening of child participation at the center and thus in line with the vision of the hospital around value-based care and innovation. At one of the hospitals, a written agreement between the researchers and the hospital was important for anchoring and giving the project legitimacy in the organization:

Yes, I think so (have had management support). Just saying that it was OK to participate. That it’s OK to write a contract about Sisom combining the perspectives of the university and the hospital. They’ve been open and accessible for...they’ve seen it as a development (of the services).

Informant 10

Another important factor described by the professionals was that their colleagues were interested and had a positive attitude toward implementing Sisom at the center. The professionals experienced that their colleagues were curious about how Sisom can support their work with child-centered care at an organizational level. The colleagues were also interested in knowing what the output was from children’s usage of Sisom and anticipated it would provide important information for the care team meetings and medical rounds at the center:

...I’ve not noticed any resistance to this. There hasn’t been any. Not among the health care staff or in our own organization...there’s been more of a curiosity about what we’ve been doing.

Informant 6

A frustration that only a few professionals at each center were practically involved in the implementation of Sisom was spoken of. Those expressing this wished that Sisom had been available to all professionals allowing its use in all situations at the center to fully support the children in their care. With only a limited number of professionals working with Sisom, the feeling at the center was that some counselors/nurses knew things about the child that other professionals would have liked to know.

#### Alignment With Working Methods and Culture

The common commitment among professionals to work consistently toward increasing child participation at each center and the shared value base of the professionals toward child-centered care were a contextual factor that promoted the implementation of Sisom. The professionals stated that it was the parents’ perspectives that predominated and had influence over the child’s care. There were no suitable tools for conveying the children’s perspectives on their care situation, and most of the professionals talked of a desire to have instruments that could help them in their conversations with the children. Mapping and adjusting to traditions, values, and attitudes of the workplaces was important for the implementation process. It was also described as easier to implement Sisom in a center where professionals had more control over their own time and where the child met the same professionals each time they visited the hospital. The professionals described that Sisom has an obvious place in their work:

...something that the social worker should do is to talk to the children but then there are several health centers where the social worker’s job is to talk to the parents instead. And at a health center where it is more common to do so (talk to the children) then Sisom can be even more effective and useful instrument. Because one gets access to the child...

Informant 2

A contextual barrier for the successful implementation of Sisom was a lack of time and that everyday work was in many ways interrupted by emergency events. The professionals were not always able to allocate enough time for meeting with the child because of the planning of additional meetings with other professionals during the visit at the hospital. The professionals described that they did not want to *squeeze* Sisom into their daily work if they were unable to guarantee that they could provide the prerequisites for carrying it out to a high standard for all those involved, the children and their families and the professionals themselves. Some professionals experienced that there was no space for working in a structured manner with Sisom:

...it’s difficult to implement everything...a great deal is needed for implementing different things in a health service like ours, where things happen all the time, where emergencies can dominate somewhat and you have to put aside your almanac all the time. Then a great deal is needed. A lot of involvement and commitment but also the structure that dictates how we should work.

Informant 6

#### Adaptability to Workplace Change

Reorganizations that occurred at some of the centers were described as a major obstacle in the implementation of Sisom, as well as changes in the form of some professionals who were committed to working with Sisom had ended their employment or had gone on parental leave during the implementation. Such reorganizations resulted in both the physical and structural conditions to work with Sisom as intended were altered, which resulted in the work with Sisom being less prioritized or forgotten for a period. The loss of people who had been assigned specific roles in the implementation process made it difficult to maintain continuity in the process and left more work for those who remained. Despite this, the implementation of Sisom had a high priority among the professionals as it was perceived as valuable for the care process:

...The challenge is often the continuity of those people who have got an assignment that they are to stay in their jobs, many have finished. The staff turnover rate is high. Continuity is a challenge nowadays.

Informant 10

### Implementation

A total of 3 subcategories emerged from the data: *structure, and content of the course*, *support from facilitators and researchers,* and *recruitment and reach*.

#### Structure and Content of the Course

Overall, the professionals had positive experiences of the content in the course. The sessions at the centers were carried out as planned. The professionals appreciated the dialogue during the session and found it valuable to reflect on what child participation means in general and how they currently worked with child participation at the center. It was especially important for new professionals, who did not have the same experience or knowledge about the importance of highlighting the child’s perspective in their practical work. All the professionals stated that Sisom worked as a motivator for their ambition to improve child participation in care and that they were very enthusiastic about using it. They appreciated that they were given the opportunity to practice using Sisom by themselves for a few days before beginning to use it with the children. Adjustments were made at all centers regarding the implementation of the intervention to suit their routines and purpose:

It (the course) was good, it was good to get the opportunity to discuss participation and what it can mean and how we perceived it. The exercises and being able to test and navigate in the app were also very good. Then I can think that if we had had...another session after some of us had started because we discovered that new questions arose...mostly concerning the usage.

Informant 3

#### Support From Facilitators and Researchers

The facilitator’s role differed somewhat among the included centers. At those where 4 or more professionals participated in the project, a facilitator was appointed to be responsible, motivate, follow up, and maintain overall contact with the researchers. At the centers with only 2 professionals working with Sisom, this facilitating role was not needed as they worked closely together and shared responsibility for the implementation process. Some of the first appointed facilitators were replaced by other people because of parental leave or termination of employment, which interrupted the implementation process.

In some cases, the facilitator did not work practically with Sisom. This was described as being both advantageous and disadvantageous. The advantage was that the facilitator had allocated time to be available to others and had an overview of the implementation process. The disadvantage was that the facilitator lacked personal experience from working practically with Sisom, which would have been valuable in supporting the professionals in their work. An important function of the facilitator was to deal with and support the professionals regarding issues or problems during the implementation:

encourage to have...a coordinating meeting and when necessary to be able to have direct contact with the staff who work with Sisom if it is needed. I think that it makes a great difference that they can meet each other...to get together and think about what we need to be able to do this in the best possible way. That one is forced to think and not just solve.

Informant 9

Confidence and trust in the researchers were described as promoting factors for the feasibility of the implementation. The professionals experienced transparency, a clear common agenda as well as availability and continuous coordination as important factors that contributed to their trust in the project. The design and structure of information material, questionnaires, checklists, etc, were experienced by the professionals as simplifying their use. Confidence was also created by researchers being prepared to modify the plan for the intervention and the evaluation:

I felt that there was always the possibility to ask questions.

Informant 7

#### Recruitment and Reach

The recruitment of staff to the project was based on the inclusion of those showing interest in joining the project. A total of 17 professionals were included in the courses of whom 15 worked practically with Sisom. In addition to these, 3 people ended their involvement because of sick leave, parental leave, and termination of employment. A total of 46 children at baseline and 33 children at follow-up participated in the project (Table 3).

All the children who met the inclusion criteria were asked about participation. The reason why the professionals did not reach more children at baseline was because of a limited number of planned appointments during the intervention period. Some children were excluded based on them not understanding Swedish. Most children and parents agreed to participate, and the reason for not being willing to participate was of a practical nature, having to come to the hospital more often thus requiring more time away from work and school. The reason for the drop-out at the follow-up (n=13) was that professionals and children did not have the possibility to meet each other twice or that some of the children died during the project period.

The professionals anticipated that the age range for inclusion could be extended. Younger children (below 6 years) usually have sufficient digital experience and competence to use Sisom. Similarly, older children (over 12 years) could have the opportunity to decide if Sisom could be appropriate for them or not:

It’s incredibly individual. We have thought that we have younger children who definitely could do this, and we have had those who are of an age that they are on the verge of being too old. Then, it’s just a game. But even if it is then they sit down and discuss anyway. Then, it didn’t become pointless anyway.

Informant 12

**Table 3.** Demographic characteristics of included children.

Characteristics		Baseline	Follow-up
**Sites, n (%)**			
	University hospital	33 (72)	22 (67)
	Rural hospital	13 (28)	11 (33)
**Sex, n (%)**			
	Female	24 (52)	17 (51)
**Age (years), n (%)**			
	6-9	21 (46)	12 (36)
	10-13	24 (52)	21 (64)
	Missing	1 (2)	0 (0)
**Diagnosis, n (%)**			
	Hematological conditions	5 (11)	3 (9)
	Cancer	15 (33)	12 (36)
	HIV	7 (15)	4 (12)
	Heart disease	4 (9)	4 (12)
	Neurological conditions	8 (17)	7 (21)
	Other	5 (11)	2 (6)
	Missing	2 (4)	1 (3)

### Mechanisms of Impact

A total of 2 subcategories emerged from the data, *applying Sisom in practice* and *technical and practical barriers.*

#### Applying Sisom in Practice

Sisom was mainly used when the children came on a planned follow-up appointment (30-60 min; see Multimedia Appendix 1). The professionals pointed out that it was important that the child used Sisom without being influenced by them or their parents. Some professionals and children wanted to discuss issues directly when Sisom was used. Most of them wrote the report and then started a discussion based on it, either during the same appointment or at a follow-up meeting a few weeks later. Some professionals asked the child and parents to discuss the report at home before the follow-up dialogue. The professionals usually chose to go through everything in the report from the child’s first use of Sisom but focusing on things that were good or problematic:

...then we’ve looked and as I said we’ve been with the children and brought up positive things also because that’s very important. And then we’ve talked about certain things where it doesn’t look so good. Here’s a red man, can you talk about it a little? And then we do that. It’s much easier than getting a direct question. Then, they’ve been able to talk about how they have felt.

Informant 12

The children found it easier to have the written report or the tablet as support for the dialogue, helping them to focus on the report instead of the professional. All the children wanted to use Sisom, but some children did not want to talk about the report afterward. However, the staff felt that the child thought it was nice that they had the opportunity to talk about how they feel without having to sit for a long time and talk about their situation. Overall, the children were satisfied with the conversation based on Sisom and most of them wanted to show Sisom and the printed report to their parents. The report became their own product and some children felt proud of what they had done:

I think that many also feel it’s good that mum and dad know that I actually think it’s difficult or that I’m worried because they are worried. It becomes clearer. If one has said it once, then it’s easier to say it again.

Informant 11

It was primarily the professionals who initiated what the discussion would focus on, and frequently, decisions were made based on the child's self-evaluations and described emotions in Sisom. The professionals pointed out that the child's reporting in Sisom cannot be interpreted as a measure of the child’s feelings, but rather requires a subsequent conversation to hear how the child reasons about the output in the report and to understand what the child meant and the child’s own view of their situation.

The professionals said that this way of working with Sisom requires preparation and planning on their part when it comes to charging the tablet, reserving a place where the child can use Sisom, printing the report, and then having time for a subsequent discussion.

### Technical and Practical Barriers

The most common barriers during the use of Sisom were technical and related to the prototypic nature of the version of Sisom used for the implementation, especially related to the printing of reports, bugs in the system, and swift termination of one child’s session before the onset of another. Bugs in the system were mostly related to the system freezing. Most often, children solved this by visiting another island and returning to the same question later on. However, the professionals felt that such technical disturbances disrupted the flow in the dialogue around the child’s assessments and therefore need to be minimized. That one child could start using Sisom if the previous session had not been terminated correctly, resulted in uncertainties about the validity of the report. The professionals emphasized that the trustworthiness of the data is a key for acceptance and that the results in the printed reports must be unambiguous:

It took a while to print the reports, but no big problems, I don’t think so, it was purely technical problem...yes, it was purely technical problem that I saw as possible problem, nothing else.

Informant 7

The professionals requested that Sisom should be available on the Web or for download so that the children could use Sisom at home before the meeting to increase effectiveness and to reduce waiting times at the clinic. Completing Sisom at home would also allow for more individualized use, based on individual capabilities and prerequisites.

Sisom was originally designed for children with cancer, and therefore some questions were less relevant for some children involved in this study. When a question was perceived as strange, the professionals recommended the child to skip it and move on. The children interpreted the questions based on their own situation, and the professionals thus stressed that it was important to not use the responses in the reports as absolute assessments of the children’s life situation but rather as support for a later discussion with the child:

There were questions that I had not thought of but which were still important...but still, it concerns a lot of people. And just like cardiac patients, they often have this nausea and difficulties eating and how one sees oneself in various ways and how one thinks that one’s friends see one. They’re very important questions, and I don’t think it’s obvious to think of such questions as a professional when one works.

Informant 8

There is no possibility to modulate the use of Sisom, in its current form, such as pausing and saving the results to work with the questions at different times or submitting comments or notes while responding to questions or while reviewing the report. This was described as a hindering factor as children, who might not have time or energy to complete all islands/questions at once, could not pause their work and professionals could not write notes or recommendations for their own documentation or for forwarding information to a colleague. Furthermore, the professionals saw a need to establish guidelines for how and where the reports should be saved, which actions should be taken based on specific information raised, and how to follow up on issues that were brought forward, and that was beyond the responsibility of that particular professional.

### Experiences of Effects

A total of 2 subcategories emerged from the data: *improved participation of children* and *improved child-centered care*.

#### Improved Participation of Children

The major advantage of implementing Sisom was that it helped children express themselves independently without the pressure of an adult who directly lead or guided the dialogue. The professionals spoke of there being a culture today that the professionals primarily talk to the parents and when the children reach 18 years of age they are expected to be able to take full responsibility themselves. Sisom was described as being a useful tool to give the opportunity for the children to train and acquire ability to take responsibility for providing their information, to express themselves, to discuss their life situation, and to be able to participate in making decisions on issues that concern themselves, thus preparing them for adult life. With Sisom, the children asked more questions than usually and found it pleasant to put words on experiences that they had not previously dared to talk about.

Many children wanted to protect their parents and as a consequence, did not dare to ask questions or raise issues that concern them but that could worry their parents. This both relieved difficult feelings among the parents and made parents more aware of views about their child’s experiences and feelings that they were not previously aware of:

It’s a good way...because we, we also work with it, when they become older teenagers and will be going to the doctor’s appointment by themselves, then it’s good for both parents and the child to train themselves in that it’s the child who is to talk, answer more and that the parents have to hold back and keep in the background a while during the appointment, and the child becomes more secure in talking with the doctor and us.

Informant 14

The professionals said that Sisom supported the children in comprehending and managing the context of the meeting with the professional resulting in that they both knew why and what they would talk about and that they were able to express themselves in a way that they felt comfortable with. The dedramatized questions in Sisom, which normally can be very sensitive or frightening, helped the children in processing their experiences about their life situation:

What he and his mother spoke of...is that they were very happy, satisfied, and positive. The boy said himself that he thought that it had been fun, but also that there were good questions, questions that made him think.

Informant 5

Similarly, the professional emphasized that the child became an actor and that the roles in their relationship clarified. In some cases, the format of the meeting was changed so that the child and the professional first met each other alone, and then, the parents were invited in. In this format, the child was involved in deciding what they wanted to talk about alone and together with their parents:

They participate more, just because it is their answers that are in focus during the appointment. They get more questions (from us). They are proud of their reports. They have been much more active in these appointments than they have been otherwise.

Informant 14

#### Improved Child-Centered Care

The experienced benefits of using Sisom were that the children's entire life situation was visualized, that the children’s own experiences about their situation appeared, that changes over time could be captured, and that decisions could be made with the children or based on the children’s perspectives on difficulties and needs. This led to increased participation of the children, and the professional’s occupational roles changed. Sisom was described as a facilitator to support professionals in applying a child-centered care approach through these effects.

The staff talked of previously having focused on pathogenic perspectives, such as the diagnosis, symptoms, treatment, and hospital-related issues. By applying Sisom, they experienced an increased ability to understand the child’s entire life situation. Working with Sisom also supported them in understanding the child’s resources, strengths, and things that were positive in the child’s situation. Sisom helped them in capturing and visualizing the child’s own experiences and gave a clearer picture of the child’s way of thinking and experiences of the situation. The effort and extra time spent on using Sisom were therefore experienced as an investment:

Because Sisom has a much broader approach when it also covers the home environment and how one feels at school. We are more, our material is more focused on the chronic illness itself and one’s experiences being at the hospital.

Informant 2

Through the use of Sisom, the professionals also became aware of areas in the child's life that needed attention but that would not normally be addressed based on the responsibilities of their professional role. This broadened awareness of the experiences and needs of the child changed their professional role in a way that shifted their perspective from being grounded in their professional expertise toward what the particular child communicated. They experienced that they would never have been able to ask all the questions provided in Sisom in a regular conversation, as it would have been both boring and tiresome for the child. The broad awareness of the child’s life situation provided by Sisom helped the professionals in not fractioning the child’s needs into the responsibilities of separate professional roles. This gave them a broader view and a new way of thinking about their professional role:

...We start talking about essential matters and it’s not because I’m a difficult social worker who asks questions about that sort of thing, but it’s the game that leads us on to these essential things...it’s not me who’s completing some paper but it’s something the child does and there are no secrets, these aren’t things I hide and don’t show, and then the child can take it home and look at it and talk with mum and dad...

Informant 7

On the same note, Sisom changed the spirit of the dialogue between the professional and the child, from the form of a structured questioning to a dialogue driven by curiosity. The professionals traditionally have all the power and lead the dialogue, thus reinforcing their dominant role and the role of the child as a subject being interrogated. Through the use of Sisom, they did not have to lean on their own or the parent’s interpretations of the children’s experiences and needs. Instead, Sisom helped them to provide information about completely different things—things that were important to the child and things that neither the professionals nor the parents knew. This made the professionals feel more relaxed in their dialogue with the child as they felt that Sisom had caught the children's perspective on what was needed to be discussed. This placed the conversation with the child at a deeper level, creating more space and time to talk about other things than they usually do. The professionals reflected that they now could approach the child in a different way, allowing the child to talk about the situation in their own way and thereby getting the *true* information from the child. Above all, Sisom was especially helpful in supporting ways of talking with those children who the professionals usually have difficulties in talking to and those children who only answer *yes* or *no* to direct questions. In these cases, Sisom created a distance to the actual questions, and the professional could act more like a sidekick in the *conversation* provided by the child’s interaction with Sisom. The professional’s role became less inquisitive and nosey, making the children more involved and share more information about their life situation:

There were questions that at first sight didn’t feel relevant, but when we spoke more about the situation it emerged that it was relevant for the boy. For example, questions about death. It isn’t what we primarily work with in relation to diabetes. There are no children who die because of diabetes. But he had questions and thoughts about it, and so we could talk more about it later. Most importantly, his mother got the chance to follow up his thoughts at home.

Informant 5

The use of Sisom also increased the professional’s ability to detect changes in the child’s situation over time. When they and the child compared the latter’s assessments in Sisom at different time points, the children were sometimes surprised over the change that had occurred. The professionals also said that the actions decided on from their discussion with the child were more person-centered because of improved awareness of the child’s situation and needs.

## Discussion

### Principal Findings

In this study, we used a process evaluation design [38] to study the implementation of the eHealth service Sisom at 4 pediatric care centers in Sweden. The weak evidence base for eHealth services in general can delay their implementation in practice [37]. When developing eHealth services and planning for evaluation, ongoing process evaluations are not commonly used. Furthermore, process evaluations per se are not commonly including both design and pretesting phases and evaluation of the clinical, human, social, and organizational aspects of the eHealth services following implementation [37]. Thus, this implementation study fills a knowledge gap by investigating facilitators and barriers that are of particular importance for successful implementation of an eHealth solution, particularly in the context of children’s participation in pediatric care. This study specifically contributes to a more comprehensive evidence base for the eHealth service Sisom. Previous research on Sisom has primarily focused on the design and pretesting phases whereas this study is the first one to investigate the implementation of Sisom in clinical practice. Further studies of implementation and effects will be needed as the use of Sisom is scaled up, reaching a more diverse array of users and settings for implementation.

The data presented in this study provided support for the strategy applied for the implementation of Sisom at the pediatric care centers involved. The professionals were satisfied with the anchoring of the project, with the course offered, and with the support provided by the local facilitators and the researchers during the project. Overall, they experienced that each of these aspects was supportive for introducing Sisom in daily practice. In changing and restructuring the way of working with patient participation in practice, it is crucial to consider contextual factors such as culture, resources, and priorities [42]. However, focusing only on the changing of knowledge, attitudes, and behavior of the professionals does not suffice. This is confirmed by professionals who often feel left alone in their effort to facilitate children’s participation [43]. In our study, the shared commitment and value base toward child-centered care at the health care centers were described as facilitating factors for a positive effective response to the intervention. Previous research has established that interventions that fit well with the organization’s existing culture and values are more likely to be successful [42]. The professionals involved in the introduction of Sisom emphasized that the knowledge base and underlying principles of the concept of participation discussed during the education helped them to generate enthusiasm for the intervention and to promote their capacity to successfully implement Sisom in practice at their center. Thus, the contextual factors affecting successful implementation were of a type that is traditionally expected to be hard to change in health care settings such as the values and attitudes, readiness for behavioral change, and the added value of the intervention [38,44,45]. Another facilitating factor for the implementation of the intervention was that the children, who were the intended users of Sisom, accepted the design of the solution and experienced meaningfulness in its use, which can probably be explained by the high degree of child participation in the development of Sisom [29]. However, there were a few contextual obstacles that could be important to consider for achieving successful implementation, such as workplace reorganization, changes in employment, changes in priorities for development work, and time constraints. Interesting findings in relation to this were that it was easier to implement Sisom at a center where professionals worked independently in relation to their colleagues, managing their own time and meeting the children individually. As independent professionals, they were able to plan and restructure their scheduling of meetings based on the children’s needs, individually planning the length of meetings and deciding to invite children to extra meetings for going through the reports from Sisom.

This implementation study provides several practical examples of how Sisom contributes to improvements in child participation. This is significant as most efforts to implement a child-centered perspective in pediatric health care have little or no evidence that those practices really provide opportunities for children to share their views, needs, and preferences and to participate in conversations about their own care [7,46,47]. The major benefit with the implemented intervention was that by using Sisom, the children could express themselves independently without the pressure from an adult and without the risk of being dominated by an adult. The professionals testified that it became evident that Sisom helped the children to dare to ask questions and talk about their experiences, thoughts, and feelings, and that the purpose of the dialogue with the health care professional became less vague and more relevant. The professionals experienced that Sisom increased their ability to detect changes in the child’s situation over time and to strengthen the children in being an actor in their own health care. Sisom also opened up different and further conversations within the family, and parents became more aware of their child’s experiences and feelings. Sisom also had an effect on the role of the parents in conversations with the professionals: from being someone who had a responsibility for responding on behalf of their child, to someone who actively listened to their child and provided support in communicating what was important to them in relation to their life situation.

### Limitation

Some limitations should be considered when interpreting the results. First, the recruitment of professionals to the project was limited to those having an interest in participating in the implementation. Participation based on voluntariness means that perspectives and experiences from professionals who would have been forced to take part in the implementation of Sisom are thus lacking. This setting limited the general impact the implementation had on the whole workplace and also made the project more vulnerable to changes in priorities and reorganizations at the participating centers. Second, further studies are needed in other health care settings and in contextual conditions in health care systems in other countries to strengthen the conclusions on the validity and generalizability of the implementation strategy applied here. Third, the implementation of Sisom needs to be investigated both longitudinally and in randomized controlled studies to analyze its long-term effects on organizations, professional roles, ways of working, and ultimately on children’s health outcomes.

### Conclusions

The changing nature of health care delivery from a provider-centered approach to an increasingly child-centered approach in which the children become actors in their own care is a challenging process. We believe this study represents a significant contribution to this field of research. To our knowledge, this is the first implementation study of an eHealth service aimed at strengthening children’s participation in pediatric health care. The results from the study show that it is possible to restructure health care delivery toward a child-centered approach if there is a willingness and preparedness in the organization to implement an eHealth solution with the aim of restructuring the way of working with children’s participation. Key factors for successful implementation are alignment of the solution with the values and goals of the organization, health care professionals’ beliefs in the usefulness and usability of the solution, and health care professionals’ willingness to change their professional roles promoted by the solution.

## Appendix

Multimedia Appendix 1 Description of how Sisom was applied, at baseline and at follow-up.

## Abbreviations

eHealth: electronic health
UNCRC: United Nation’s Convention of the Rights of the Child
