# Supplementary material for: Barriers and Enablers Affecting Successful Implementation of the Electronic Health Service Sisom: Multicenter Study of Child Participation in Pediatric Care
Source: J Med Internet Res. 2019 Nov 15;21(11):e14271. doi: 10.2196/14271 (PMC6884717; doi:10.2196/14271)
Supplement: Multimedia Appendix 1 [file jmir_v21i11e14271_app1.pdf]

Supplementary Table 1. Description of how Sisom was applied, at baseline and at follow up.

		Baseline (n=46)	Follow-up (n=33)
Type of appointment	Treatment	7 (15 %)	4 (12 %)
	New appointment	3 (6.5 %)	0 (0 %)
	Follow-up	24 (52.5 %)	24 (73 %)
	Sampling	6 (13 %)	0 (0 %)
	Other not specified	5 (11 %)	4 (12 %)
	Missing	1 (2 %)	1 (3 %)
Professionals participating at the appointment	Nurse/Counsellor	31 (67,5 %)	23 (69 %)
	2-3 professionals*	14 (30,5 %)	9 (27 %)
	Missing	1 (2 %)	1 (3 %)
Roles present when using Sisom	Only the child	5 (11 %)	10 (30 %)
	Parents	14 (30 %)	7 (21 %)
	Professionals	19 (41 %)	10 (30 %)
	Parents & professionals	7 (15 %)	4 (12 %)
	Missing	1 (2 %)	2 (6 %)
Time used for the meeting	<30 minutes	17 (37 %)	12 (36 %)
	30-60 minutes	23 (50 %)	17 (51 %)
	>60 minutes	4 (9 %)	1 (3 %)
	Missing	2 (4 %)	3 (9 %)
Sisom report used as the basis for the dialogue during the meeting	Yes	29 (63 %)	27 (89 %)
	No	16 (35 %)	5 (15 %)
	Missing	1 (2 %)	1 (3%)
Choosing which parts in the report to talk about	The child initiated	8 (17 %)	7 (21 %)
	The professional initiated	15 (33 %)	13 (39 %)
	The parents initiated	1 (2 %)	0 (0%)
	The child and the professional initiated together	9 (19 %)	6 (18 %)
	The professional and the parent initiated together	0 (0 %)	1 (3 %)
	No dialogue was done	1 (2 %)	1 (3 %)
	Missing	12 (26 %)	5 (15 %)
Action or decision taken based on the report and the following meeting	Yes	18 (39 %)	9 (27 %)
	No	27 (59 %)	22 (67 %)
	Missing	1 (2 %)	2 (6 %)

* nurse, doctor, counsellor or other
